# Association of sweetened beverages consumption with all-cause mortality risk among Dutch adults: the Lifelines Cohort Study (the SWEET project)

**DOI:** 10.1007/s00394-022-03023-6

**Published:** 2022-10-21

**Authors:** Novita D. Naomi, Elske M. Brouwer-Brolsma, Marion E. C. Buso, Sabita S. Soedamah-Muthu, Joanne A. Harrold, Jason C. G. Halford, Anne Raben, Johanna M. Geleijnse, Edith J. M. Feskens

**Affiliations:** 1 Division of Human Nutrition and Health, Wageningen University and Research, P.O. Box 17, 6700 AA Wageningen, The Netherlands; 2 Department of Medical and Clinical Psychology, Center of Research on Psychological Disorders and Somatic Diseases (CORPS), Tilburg University, Tilburg, The Netherlands; 3 Institute for Food, Nutrition and Health, University of Reading, Reading, UK; 4 Department of Psychology, University of Liverpool, Liverpool, UK; 5 School of Psychology, University of Leeds, Leeds, UK; 6 Department of Nutrition, Exercise, and Sports, Faculty of Science, University of Copenhagen, Copenhagen, Denmark; 7 Clinical Research, Copenhagen University Hospital-Steno Diabetes Center Copenhagen, Herlev, Denmark

**Keywords:** Dutch adults, Non-nutritive sweeteners, Artificial sweeteners, Soft drink, Juice, Death

## Abstract

**Purpose:**

Examined associations between sugar-sweetened beverages (SSB), low/no-calorie beverages (LNCB), and fruit juice (FJ) consumption and all-cause mortality in Dutch adults.

**Methods:**

Data of 118,707 adults participating (mean age = 45 years; 60% was women) the Lifelines Cohort Study were prospectively analyzed. Dietary intake was assessed using a validated food-frequency questionnaire. Participants’ vital status was followed-up until February 2022 via the National Personal Records Database. Associations between beverages of interest and all-cause mortality risk were investigated using restricted cubic spline and Cox proportional hazard regression analyses, including substitution analyses. Models were adjusted for demographics, lifestyle, and other dietary factors.

**Results:**

During follow-up (median = 9.8 years), a total of 2852 (2.4%) deaths were documented. Median (IQR) of SSB, LNCB, and FJ consumption were 0.1 (0.0–0.6), 0.1 (0.0–0.6), and 0.2 (0.0–0.6) serving/day, respectively. Dose–response analyses showed linear associations between SSB, LNCB, and FJ consumption and mortality risk. For each additional serving of SSB and LNCB, HRs of all-cause mortality risk were 1.09 (95% CI 1.03–1.16) and 1.06 (95% CI 1.00–1.11). Replacing SSB with LNCB showed a nonsignificant association with a lower mortality risk, particularly in women (HR 0.91, 95% CI 0.81–1.01). Finally, an inverse association between FJ and all-cause mortality was observed at moderate consumption with HR of 0.87 (95% CI 0.79–0.95) for > 0–2 servings/week and HR of 0.89 (95% CI 0.81–0.98) for > 2–< 7 servings/week when compared to no consumption.

**Conclusions:**

Our study showed adverse associations between SSB consumption and all-cause mortality. Replacing SSB with LNCB might be associated with lower mortality risk, particularly in women. Moderate intake of FJ was associated with lower all-cause mortality risk.

**Supplementary Information:**

The online version contains supplementary material available at 10.1007/s00394-022-03023-6.

## Introduction

An unhealthy diet has recently been shown to account for around 11 million deaths worldwide [1]. High sugar consumption has been suggested to play a substantial role in this by adversely affecting risks of cardiometabolic disease [2–5]. Consequently, sugar is increasingly replaced by low/no-calorie sweeteners to reduce caloric content while maintaining sweetness [6]. Yet, evidence on the impact of sugar-sweetened beverages (SSB) and low/no-calorie beverages (LNCB) on mortality risk remains scarce and inconclusive. Recent dose–response meta-analyses showed significant positive associations between SSB and LNCB consumption and all-cause mortality risk [4, 7, 8], but also showed moderate to high heterogeneity.

The observed heterogeneity may relate to inconsistencies in terms of the definition of SSB. Whereas some research groups include fruit juices (FJ) in the definition of SSB, others study FJ as a separate food group. In terms of composition, SSB mainly consists of liquids sweetened with various forms of added sugars, FJ contains sugars as well as polyphenols, vitamins, and minerals. Besides, as recent data suggest a potential J-shaped association between LNCB and all-cause mortality [7], and between 100% FJ and various cardiometabolic disease risks [9, 10], there is a need to investigate the non-linearity of the associations. Finally, although SSB is often replaced by other beverages, substitution modelling of these replacements is scarce. Recent studies observed an inverse association between replacing SSB with LNCB and all-cause mortality [11, 12], whereas another study showed no association with coronary deaths [13].

All in all, the varying results as displayed above highlight the complexity of the study on SSB and LNCB in relation to health. Therefore, we prospectively studied the associations, as well as the theoretical substitution, between SSB, LNCB, and FJ consumption and all-cause mortality risk among Dutch adults.

## Methods

### Study population

The SWEET project is a European Union-funded project that aims to examine the use of sweeteners and sweetness enhancers, including risks and benefits of using them to replace sugar toward health, safety, and sustainability contexts (www.sweetproject.eu). This current study using data from the Lifelines Cohort Study was conducted as part of the investigation of the associations between sugar and sweeteners consumption and cardiometabolic health using data from various population-based studies.

Lifelines is a multi-disciplinary prospective population-based cohort study examining in a unique three-generation design the health and health-related behaviors of 167,729 persons living in the North of The Netherlands [14, 15]. It employs a broad range of investigative procedures in assessing the biomedical, socio-demographic, behavioral, physical, and psychological factors that contribute to the health and disease of the general population, with a special focus on multi-morbidity and complex genetics. Participants were recruited between 2006 and 2013, and will be followed for over 30 years. Those with serious psychiatric or physical disease, limited life expectancy (< 5 years), or inadequate knowledge of the Dutch language were not eligible. Every 1.5 years, a follow-up questionnaire will be administered and every 5 years an on-site physical assessment will be scheduled. For current analyses, 152,728 participants aged ≥ 18 years were included. After exclusion of participants with missing dietary data (*n* = 8633), unreliable energy intakes (men with energy intake < 800 or > 4000 kcal/day or women with energy intake < 500 or > 3500 kcal/day) [16] (*n* = 15,483), or missing covariate data (*n* = 9905), *n* = 118,707 participants remained eligible for current analyses (flowchart in Supplemental Fig. 1). Lifelines was conducted under principles of the Declaration of Helsinki and the research code University Medical Center Groningen (UMCG) and has been approved by The Medical Ethical Review Committee of the University Medical Center in Groningen (No. 2007/152). All participants provided written informed consent before participation.

### Assessment of dietary intake

At baseline, dietary intake was assessed using a validated 110-items semiquantitative Food Frequency Questionnaire (FFQ), where the previous month served as the reference period [17]. The average daily nutrient intake was calculated by multiplying consumption frequency by portion size and nutrient content in grams as indicated in the Dutch food composition table (2011) [18]. SSB covered all soft drinks or lemonades with sugar, such as coke and orange-flavored soft drinks, or lemonade with syrup. LNCB referred to all diet soft drinks or lemonades where sugar was replaced by low/non-calorie sweeteners. Coffee or tea with sugar or sweetened dairy drinks were not included in SSB and LNCB definitions. FJ covered mainly pasteurized juice, i.e., apple juice and orange juice. SSB, LNCB, and FJ consumption were analyzed per serving of 150 ml, i.e., the smallest serving size in Europe [19], as well as in categories of consumption: no consumption, > 0–2 servings/week (servings/week), > 2–< 7 servings/week, and ≥ 1 serving/day (servings/day).

### Assessment of outcome

Vital status of participants was obtained through passive monitoring via linking the Lifelines data to the national Personal Records Database. Information on the month and year of all-cause mortality was recorded up to February 2022 for current analysis, which resulted in a median follow-up time of 9.8 years (interquartile range [IQR] 8.9–10.7).

### Assessment of covariates

Baseline data on medical history, demographic, anthropometric, and lifestyle factors were collected using self-administered questionnaires. Educational level was categorized as low (primary education or less), moderate (lower or preparatory vocational education, lower general secondary education, intermediate vocational education or apprenticeship, or higher general secondary education or pre-university secondary education), or high education (higher vocational education or university). Smoking status was reported in four categories as never, former, current (< 10/day), or current (≥ 10/day). Physical activity was assessed using the validated Short Questionnaire to ASsess Health-enhancing physical activity (SQUASH) [20] and the Activity Questionnaire for Adults and Adolescents (AQuAA) [21]. Physical activity was reported in Metabolic equivalent (MET)-minutes/week for moderate level activity and in minutes/week for sedentary behavior (TV watching). Alcohol consumption was assessed using the FFQ from which ethanol consumption was calculated and categorized as 0, > 0 to  ≤ 10, > 10 to  ≤ 20, or > 20 g/day. Body weight (kg) and height (cm) were measured with SECA 761 scale and SECA 222 stadiometer, respectively, and measures were rounded to the nearest 0.5 cm and 0.1 kg for height and weight, respectively. Body mass index (BMI) was calculated by dividing weight (kg) by square height (m^2^).

### Statistical analysis

To first investigate the dose–response associations between SSB, LNCB, and FJ consumption and all-cause mortality risk, restricted cubic spline analyses were performed [22]. The fit of the spline model was tested against a linear model with a likelihood-ratio test. Associations between SSB, LNCB, and FJ consumption and all-cause mortality risk were then investigated using Cox proportional hazard regression analyses, resulting in hazard ratios (HRs) with their 95% confidence interval (CI). Survival time (months) was calculated by subtracting the date of baseline measurement (month and year) from the time of death (month and year) or end of follow-up (February 2022), whichever came first. To investigate the association with all-cause mortality when replacing SSB with an equivalent amount of LNCB or FJ, theoretical substitution analyses were conducted by means of a leave-one-out model [16]. This model included the sum of SSB, LNCB, and FJ consumption (in serving/day) as one variable, followed by beverages defined as the replacement, as well as all other covariates as modelled in Cox proportional analyses. For all above analyses, models were adjusted for age, sex (model 1), education level, alcohol consumption, smoking status, moderate physical activity, sedentary behavior, BMI (model 2), grains, potatoes, vegetables, fruit, meat and processed meat, coffee, tea, legumes, nuts, fats and oils, sugary foods, mutual adjustment for the other beverages (SSB, LNCB, and fruit juice) (servings/day), and total energy intake (model 3). To explore the presence of reverse causation, sensitivity analysis was conducted where the first 2 years of follow-up were omitted and thus excluding all new cases identified during that period from the analyses (*n* = 249). Stratification analyses were performed to examine whether the HR of all-cause mortality differed across strata of sex (men or women), education level (low/moderate or high), BMI categories (< 25 or ≥ 25), and the presence of diseases (prevalence diabetes or history of hypertension, hypercholesterolemia or cardiovascular disease [CVD]) (yes or no). All analyses were performed using R 4.0.2 and RStudio 2022.02.0.

## Results

Participants had a mean ± SD age of 45 ± 13 years (60% women; 55% had BMI ≥ 25; 21% current smokers) (Table 1). Median (IQR) of SSB (37% non-consumers), LNCB (44% non-consumers), and FJ (24% non-consumers) consumption were 0.1 (0.0–0.6), 0.1 (0.0–0.6), and 0.2 (0.0–0.6) servings/day, respectively. During a median (IQR) follow-up period of 9.8 [8.9–10.7] years, a total of 2852 (2.4%) deaths were documented. Compared to men, women were less likely to be physically active or smokers, and less often have BMI ≥ 25 or hypercholesterolemia. Men and women with higher SSB consumption tend to be younger, lower educated, smokers, less physically active, and have a higher energy intake; but they are less likely to have BMI ≥ 25, diabetes, history of hypercholesterolemia, hypertension, or history of CVD (Supplemental Table 2). Men and women with higher LNCB consumption were younger, lower educated, less physically active, and more likely to be smokers, or to have BMI ≥ 25 or diabetes (Supplemental Table 3). Higher SSB and LNCB consumption were associated with lower consumption of vegetables, fruits, and legumes, but with higher consumption of meat and processed meat. Higher SSB consumption was also associated with higher consumption of FJ and sugary food. Similar patterns were observed when comparing participant characteristics of those with higher vs lower FJ consumption (Supplemental Table 4).

**Table 1:** Baseline characteristics of 118,707 participants of Lifelines

Characteristics	All (*n* = 118,707)	Sex categories^a^	
		Men (*n* = 47,943)	Women (*n* = 70,764)
Age, years	45 ± 13	46 ± 13	44 ± 13
Education			
Low	4897 (4)	1760 (4)	3137 (4)
Moderate	77,105 (65)	30,367 (63)	46,738 (66)
High	36,705 (31)	15,816 (33)	20,889 (30)
Smoking status			
Never	54,985 (46)	20,711 (43)	34,274 (49)
Former	39,342 (33)	16,491 (35)	22,851 (32)
Current < 10/day	11,088 (9)	4801 (10)	6287 (9)
Current ≥ 10/day	13,292 (11)	5940 (12)	7352 (10)
Moderate physical activity, MET-min/week	1611 [751–2904]	1728 [810–3134]	1536 [735–2748]
Sedentary behavior, min/week	840 [630–1260]	840 [630–1260]	910 [630–1260]
Alcohol use			
0 g/day	3278 (3)	744 (2)	2534 (4)
> 0 to ≤ 10 g/day	84,760 (71)	28,315 (59)	56,445 (80)
> 10 to ≤ 20 g/day	22,269 (19)	12,509 (26)	9760 (14)
> 20 g/day	8400 (7)	6375 (13)	2025 (3)
Prevalent diabetes	2967 (3)	1372 (3)	1595 (2)
Hypercholesterolemia	16,146 (14)	8335 (17)	7811 (11)
Hypertension	26,208 (22)	9820 (21)	16,388 (23)
History of CVD	2825 (2)	1590 (3)	1235 (2)
BMI, kg/m^2^	26.0 ± 4.3	26.4 ± 3.7	25.8 ± 4.7
≥ 25	65,166 (55)	30,137 (63)	35,029 (50)
SSB, serving/day	0.1 [0.0–0.6]	0.3 [0.0–0.9]	0.1 [0.0–0.5]
LNCB, serving/day	0.1 [0.0–0.6]	0.1 [0.0–0.6]	0.1 [0.0–0.6]
FJ, serving/day	0.2 [0.0–0.6]	0.2 [0.0–0.7]	0.1 [0.0–0.6]
Total energy, kcal/day	2027 ± 576	2328 ± 591	1823 ± 466

Value are in means ± SDs for normally distributes variables, medians [25th, 75th] for nonnormally distributed variables or *n* (%) for categorical variable

*CVD* cardiovascular disease, *FJ* fruit juice, *LNCB* low-calorie sweetened beverages, *MET* metabolic equivalent task, *SBB* sugar-sweetened beverages

^a^Comparisons of characteristics between men and women were tested using ANOVA, Kruskal–Wallis or chi-squared tests as applicable. All were statistically significant (*P* =  < .001)

**Fig. 1:**
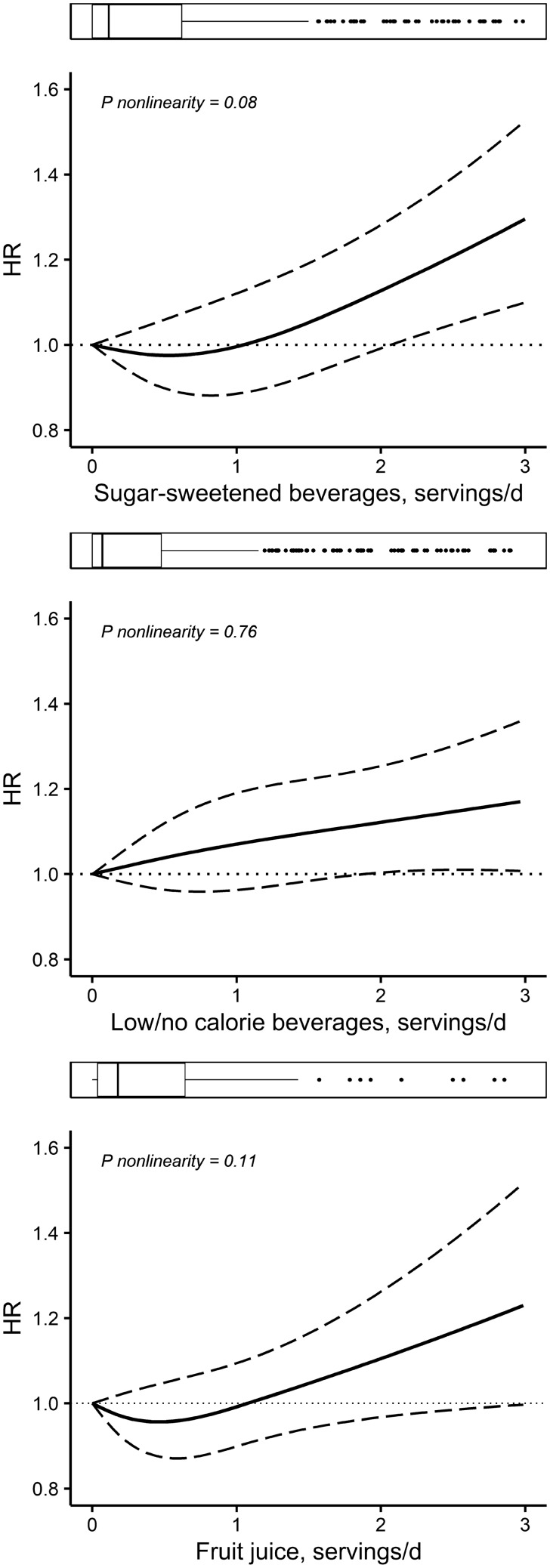
Dose–response associations between sugar-sweetened beverages, low/no-calorie beverages and fruit juice consumption and all-cause mortality in Lifelines. Solid lines are risk estimates evaluated using restricted cubic splines indicating the shape of the association in the continuous scale Three knots with 0 serving/day as a reference value were placed. Beverages consumptions was truncated at 3 serving/day. Areas between dash lines indicate 95% confidence intervals. Model was adjusted for age, sex, education level, alcohol consumption, smoking status, moderate physical activity, sedentary behavior, baseline BMI, grain, potatoes, vegetables, fruit, meat and processed meat, coffee, tea, legumes, nuts, fats and oils, sugary foods, mutual adjustment for other beverages (sugar-sweetened beverages, low/no-calorie beverages and fruit juice), and energy intake

Dose–response analysis did not reveal strong evidence of a nonlinear association between SSB consumption and all-cause mortality (*P* = 0.08) (Fig. 1). Linearly, each additional serving/day of SSB consumption was associated with 19% higher all-cause mortality risk (HR 1.19, 95% CI 1.13–1.25) after adjustment for age and sex (Table 2). Further adjustment for demographic, lifestyle, and dietary factors attenuated this association (HR 1.09, 95% CI 1.03–1.16). There was clear evidence for interaction between SSB consumption and sex in the association with all-cause mortality (*P* = 0.003). Sex-stratified analyses indicated a more pronounced association in women (HR 1.15, 95% CI 1.05–1.25) than in men (HR 1.06, 95% CI 0.99–1.14), which is also visually displayed in the stratified dose–response analysis (Supplemental Fig. 2). Sensitivity analysis by omitting the first 2 years of follow-up did not alter the associations of SSB with all-cause mortality in the total cohort (HR 1.07, 95% CI 1.00–1.13) (Supplemental Table 5).

**Table 2:** Association of SSB, LNCB, and FJ consumption with all-cause mortality risk in 118,707 participants of Lifelines

	HR (95% CI) by categories of beverages consumption				HR (95% CI) for each servings/day increment	*P* for interaction
	No consumption	> 0–2 servings/week	> 2- < 7 servings/week	≥ 1 servings/day		
SSB						
*n*	43,927	29,706	25,595	19,479	118,707	
Event (%)	1471 (3.3)	655 (2.2)	428 (1.7)	298 (1.5)	2852 (2.4)	
Model 1	1 (ref)	0.88 (0.81–0.97)	0.96 (0.86–1.08)	1.37 (1.21–1.56)	1.19 (1.13–1.25)	
Model 2	1 (ref)	0.91 (0.83–1.00)	0.96 (0.86–1.06)	1.23 (1.08–1.40)	1.12 (1.07–1.18)	
Model 3	1 (ref)	0.91 (0.83–1.01)	0.93 (0.83–1.04)	1.12 (0.97–1.29)	1.09 (1.03–1.16)	
Men^a^						0.003
*n*	13,313	12,155	11,992	10,483	47,943	
Event (%)	713 (5.4)	356 (2.9)	253 (2.1)	194 (1.9)	1516 (3.2)	
Model 3	1 (ref)	0.82 (0.72–0.94)	0.85 (0.73–0.99)	1.09 (0.91–1.31)	1.06 (0.99–1.14)	
Women^a^						
*n*	30,614	17,551	13,603	8996	70,764	
Event (%)	758 (2.5)	299 (1.7)	175 (1.3)	104 (1.2)	1336 (1.9)	
Model 3	1 (ref)	1.02 (0.89–1.17)	1.03 (0.87–1.23)	1.15 (0.91–1.44)	1.15 (1.05–1.25)	
LNCB						
*n*	52,375	23,842	25,494	16,996	118,707	
Event (%)	1471 (2.8)	565 (2.4)	487 (1.9)	329 (1.9)	2852 (2.4)	
Model 1	1 (ref)	0.86 (0.78–0.95)	0.94 (0.85–1.05)	1.22 (1.08–1.38)	1.10 (1.04–1.15)	
Model 2	1 (ref)	0.90 (0.82–0.99)	0.95 (0.86–1.06)	1.14 (1.01–1.29)	1.06 (1.00–1.11)	
Model 3	1 (ref)	0.93 (0.84–1.03)	0.96 (0.86–1.07)	1.15 (1.02–1.30)	1.06 (1.00–1.11)	
Men^a^						0.96
*N*	22,027	8806	10,238	6872	47,943	
Event (%)	770 (3.5)	288 (3.3)	274 (2.7)	184 (2.7)	1516 (3.2)	
Model 3	1 (ref)	0.93 (0.81–1.06)	0.99 (0.86–1.14)	1.19 (1.01–1.41)	1.06 (0.99–1.13)	
Women^a^						
*n*	30,348	15,036	15,256	10,124	70,764	
Event (%)	701 (2.3)	277 (1.8)	213 (1.4)	145 (1.4)	1336 (1.9)	
Model 3	1 (ref)	0.93 (0.81–1.07)	0.91 (0.78–1.07)	1.07 (0.89–1.29)	1.04 (0.97–1.12)	
FJ						
*n*	28,346	38,053	39,088	13,220	118,707	
Event (%)	963 (3.4)	817 (2.1)	825 (2.1)	247 (1.9)	2852 (2.4)	
Model 1	1 (ref)	0.79 (0.72–0.87)	0.84 (0.76–0.92)	1.13 (0.98–1.31)	1.08 (1.01–1.15)	
Model 2	1 (ref)	0.86 (0.78–0.95)	0.91 (0.83–1.00)	1.17 (1.02–1.35)	1.08 (1.02–1.16)	
Model 3	1 (ref)	0.87 (0.79–0.95)	0.89 (0.81–0.98)	1.10 (0.95–1.27)	1.05 (0.98–1.12)	
Men^a^						0.07
*n*	10,881	14,263	16,445	6354	47,943	
Event (%)	523 (4.8)	430 (3.0)	424 (2.6)	139 (2.2)	1516 (3.2)	
Model 3	1 (ref)	0.85 (0.75–0.97)	0.80 (0.71–0.92)	1.07 (0.88–1.30)	1.00 (0.91–1.10)	
Women^a^						
*n*	17,465	23,790	22,643	6866	70,764	
Event (%)	440 (2.5)	387 (1.6)	401 (1.8)	108 (1.6)	1336 (1.9)	
Model 3	1 (ref)	0.88 (0.76–1.01)	0.99 (0.86–1.13)	1.10 (0.88–1.38)	1.09 (0.98–1.20)	

Model 1: adjusted for age, sex; model 2: model 1 with additionally adjusted for education level, alcohol consumption, smoking status, moderate physical activity, sedentary behavior, baseline BMI; model 3: model 2 with additionally adjusted for consumptions of grain, potatoes, vegetables, fruit, meat and processed meat, coffee, tea, legumes, nuts, fats and oils, sugary foods, mutual adjustment for other beverages (SSB, LNCB, and fruit juice), and energy intake

*FJ* fruit juice, *LNCB* low-calorie sweetened beverages, *SBB* sugar-sweetened beverages

^a^*P* value for interaction with sex was calculated in each serving/day increment for SSB and LNCB and using categorical model for FJ

Dose–response analysis suggested a linear association between LNCB consumption and higher all-cause mortality risk (*P* = 0.76) (Fig. 1). Each additional serving/day of LNCB consumption was associated with a 10% higher all-cause mortality risk (HR 1.10, 95% CI 1.04–1.15) after adjustment for age and sex (Table 2). This association was slightly attenuated in the fully adjusted model (HR 1.06, 95% CI 1.00–1.11). Omitting the first 2 years of follow-up period did not change the associations (Supplemental Table 5). However, when excluding participants with disease history, no association was observed between LNCB and all-cause mortality risk (Supplemental Table 6).

Dose–response analysis did not show a strong evidence of nonlinear association between FJ consumption and all-cause mortality risk in the total population (*P* = 0.11) (Fig. 1). In the linear analysis, no association between each additional serving/day of FJ and all-cause mortality was observed (HR 1.05, 95% CI 0.98–1.12) (Table 2). However, Cox proportional hazard analysis across categories of FJ consumption showed an inverse association with all-cause mortality at moderate consumption levels (HR 0.87, 95% CI 0.79–0.95 in > 0 to 2 servings/week consumers and HR 0.89, 95% CI 0.81–0.98 in > 2–< 7 servings/week consumers), but not at higher consumption levels (HR 1.10, 95% CI 0.95–1.27), when compared to no consumption (Table 2). After omitting the first 2 years of the follow-up, associations were essentially the same (Supplemental Table 5).

When SSB was replaced with an equal amount of LNCB, the HR for all-cause mortality was 0.97 (95% CI 0.90–1.04) (Table 3). In stratified analyses, an nonsignificant inverse association was observed in women (HR 0.91, 95% CI 0.81–1.01), but not in men (HR 0.99, 95% CI 0.90–1.10). However, the analysis did not suggest a significant interaction with sex (*P* = 0.97). When replacing SSB with the same amount of FJ, no solid evidence of an association with all-cause mortality was observed (HR 0.96, 95% CI 0.88–1.05).

**Table 3:** Associations for all-cause mortality when replacing SSB with an equivalent amount of LNCB or FJ in 118,707 participants of Lifelines

	HR (95% CI) for each servings/day replacement^a^
SSB with LNCB	
All	0.97 (0.90–1.04)
Men	0.99 (0.90–1.10)
Women	0.91 (0.81–1.01)
SSB with FJ	
All	0.96 (0.88–1.05)
Men	0.94 (0.83–1.07)
Women	0.94 (0.82–1.09)

Adjusted for total beverages, mutual adjustment for other beverages (LNCB or FJ), age, education level, alcohol consumption, smoking status, moderate physical activity, sedentary behavior, baseline BMI, grain, potatoes, vegetables, fruit, meat and processed meat, coffee, tea, legumes, nuts, fats and oils, sugary foods, and energy intake

*FJ* fruit juice, *LNCB* low-calorie sweetened beverages, *SBB* sugar-sweetened beverages

^a^*P* for interaction was 0.96 for SSB replaced with LNCB and 0.05 for SSB replaced with FJ

Finally, stratified analyses did not indicate evidence for effect modification by BMI, age, or educational level in any of the associations under study.

## Discussion

In this study, each additional serving/day of SSB was associated with a 9% higher all-cause mortality risk, which was most pronounced in women. For LNCB, each additional serving/day was associated with 6% higher all-cause mortality risk, but replacing SSB by LNCB was likely to be associated with a lower all-cause mortality risk particularly in women. Finally, although there is no strong evidence of nonlinear association, an inverse association between FJ and all-cause mortality was observed at moderate consumption (< 1 serving/day), but not in higher consumption levels.

Positive associations between SSB and all-cause mortality risk have also been reported in recent meta-analyses of cohort studies [4, 7, 8, 23], where particularly the largest studies support our findings [11, 12, 24–27]. A pooled prospective analysis of the Nurses’ Health Study and Health Professional Follow-up Study [12] yielded a 7% higher mortality risk for each additional serving/day. Comparing the highest consumption group (≥ 2 servings/day) vs reference (< 1/month) also showed a positive association that was more pronounced in women than in men (HR 1.25, 95% CI 1.16–1.34 vs 1.12, 95% CI 1.00–1.26) [12]. The sex-specific difference might be explained by physiologic differences between men and women, e.g., sex hormones and lipid profile, but further research is warranted [28–30]. However, not all studies are in line with our findings, which might be due to differences in study methodology, such as in SSB definition (i.e., including juice, added sugar) and age of included participants [27, 31–35].

Our data showed an association between LNCB and higher all-cause mortality risk. A recent meta-analysis by Pan et al. [8] including eight prospective cohort studies, also showed a higher all-cause mortality with LNCB consumption (HR 1.04, 95% CI 1.00–1.09 for each 250 ml/day). In line with our findings, several studies in this meta-analysis reported attenuation of positive associations after excluding those with diseases history, which may indicate the presence of reverse causation [12, 25]. We also identified other potential signs of reverse causation in our study, i.e., participants with higher LNCB consumption tended to have BMI ≥ 25 than participants with lower consumption. People might have switched to healthier diet once diagnosed with relevant risk factors to control their health, i.e., BMI. Taking this all together, our results need to be interpreted with caution.

We observed an inverse association between FJ and all-cause mortality at moderate intake level of < 1 serving/day, but not at higher consumption levels, when compared to no consumption. Similarly, the UK Biobank study [25] (*n* = 161,415) showed a 9% lower mortality risk when comparing those consuming ≤ 1 serving/day of FJ with non-consumers, which was not observed among those consuming > 1 serving/day. However, in the UK Biobank study, the association in the moderate consumption (≤ 1 serving/day) group disappeared after adjustment for diet quality. A recent meta-analysis of two prospective data by D’Elia et al. [10] suggested a nonlinear association between low to moderate 100% FJ consumption with stroke (≤ 200 ml/day) and CVD events (≤ 170 ml/day) (reference: no consumption), while no significant association was observed in the higher consumption category. In addition, Khan et al. [9] also demonstrated an inverse association between FJ consumption below 150 ml and CVD incidence, which was not present at higher consumption.

To date, limited studies investigated the replacement of SSB with LNCB or FJ and mortality risk [11–13]. In the present study, we observed nonsignificant 9%lower all-cause mortality risk when replacing SSB with LNCB in women, which is similar to previous findings in total population (HR 0.93, 95% CI 0.87–1.00) [11]. Pooled analysis of the Nurses’ Health Study and Health Professional Follow-up Study showed a 4% lower risk of all-cause mortality when replacing SSB with LNCB (HR 0.96, 95% CI 0.94–0.98) [12]. Various experimental studies also showed beneficial effects of replacing SSB with LNCB, especially with respect to weight loss or weight maintenance and some cardiometabolic profiling, i.e., body fat percentages and intrahepatocellular lipid [36]. In terms of replacing SSB with FJ, we observed no association with all-cause mortality, which is also in line with the previous study [11].

Adverse association between SSB and mortality risk can be explained by several biological mechanisms. SSB consumption may induce hepatic de novo lipogenesis, hyperuricemia, and insulin resistance by the high fructose content [37]. SSB consumption is also associated with decreased satiety and insufficient adjustment of energy reduction after liquid calories consumption compared to isocaloric solid food, which subsequently contribute to weight gain [5]. LNCB has been suggested to disturb the reward system, sweetness perception, and induce gut microbiota dysbiosis, which may lead to metabolic homeostasis disruption and insulin resistance [38–40]. However, evidence supporting these suggested undesirable effects is limited and more human experimental study is still needed [41]. Underlying mechanisms explaining the association between juice consumption and mortality risk are also not yet clear. FJ may contain a high amount of antioxidants (i.e., polyphenols) and other bioactive components (i.e., vitamin and mineral) [9, 10, 42] that can be beneficial for health, but food processing may affected antioxidant content [43]. Like SSB, FJ also has a high sugar content, which may counteract its benefits at higher consumption [9, 44].

Strengths of this study include its large sample size and long follow-up period, allowing for well-powered stratified analyses conducted in a unique three-generation design. Moreover, our study population is representative of the Dutch population in terms of socioeconomic, lifestyle, and disease prevalence [15]. The theoretical substitution analysis is another strength of this study as it provides insight into public health implications of using LNCB as an alternative for SSB. One of the limitations of our study is that we were unable to distinguish between various types (brands) of LNCB and FJ, which requires further study with respect to their potential differential impact on cardiometabolic health and mortality risks. In addition, dietary consumption was only assessed at baseline while repeated dietary assessment over time could have further reduced the potential of reverse causality and provided more precise risk estimates [12]. Furthermore, deaths due to non-chronic conditions might have attenuated all-cause mortality. However, in the Netherlands, the proportion of deaths in the Netherlands due to other than non-communicable disease was low (< 7%) [45, 46]. This proportion was higher (16%) in 2020 due to COVID-19, which was mainly among older people that also often suffered from co-existing non-communicable disease. Therefore, major attenuation of our findings due to death other than chronic conditions was unlikely. Finally, although we were able to account for a wide range of confounders, residual confounders cannot be excluded.

In conclusion, our findings suggest a positive association between SSB consumption and all-cause mortality risk, which was more pronounced in women than in men. Replacing SSB with LNCB might be associated with a lower all-cause mortality risk, particularly in women. Finally, an inverse association with all-cause mortality risk was observed at moderate consumption of FJ.

## Supplementary Information

Below is the link to the electronic supplementary material.

Supplementary file1 (PDF 619 kb)
