# Supplementary material for: Association of sweetened beverages consumption with all-cause mortality risk among Dutch adults: the Lifelines Cohort Study (the SWEET project)
Source: Eur J Nutr. 2022 Oct 21;62(2):797–806. doi: 10.1007/s00394-022-03023-6 (PMC9589708; doi:10.1007/s00394-022-03023-6)
Supplement: Supplementary file 1 — Supplementary file1 (PDF 619 kb) [file 394_2022_3023_MOESM1_ESM.pdf]

Supplemental Fig. 1 Flow chart of the exclusion criteria in Lifelines.

Supplemental Fig. 2 Dose-response associations between sugar-sweetened beverages, low/no calorie beverages and fruit juice consumption and all-cause mortality in men and women in Lifelines.

Supplemental Table 1 Dietary consumption of 118,707 participants of Lifelines

Supplemental Table 2 Baseline characteristics of 118,707 participants of Lifelines by categories of SSB consumption and stratified by sex

Supplemental Table 3 Baseline characteristics of 118,707 participants of Lifelines by categories of LNCB consumption and stratified by sex

Supplemental Table 4 Baseline characteristics of 118,582 participants of Lifelines by categories of FJ consumption and stratified by sex

Supplemental Table 4 Association between SSB, LNCB, and FJ consumption and all-cause mortality in 118,458 participants of Lifelines after omitting the first 2 years of follow-up

Supplemental Table 6 Association between SSB, LNCB, and FJ consumption and all-cause mortality in 118,707 participants of Lifelines when stratified by categories of BMI, education level, and prevalence of diseases

Supplemental Table 7 Association between SSB, LNCB, and FJ consumption and all-cause mortality among daily consumers of SSB, LNCB and FJ in Lifelines.

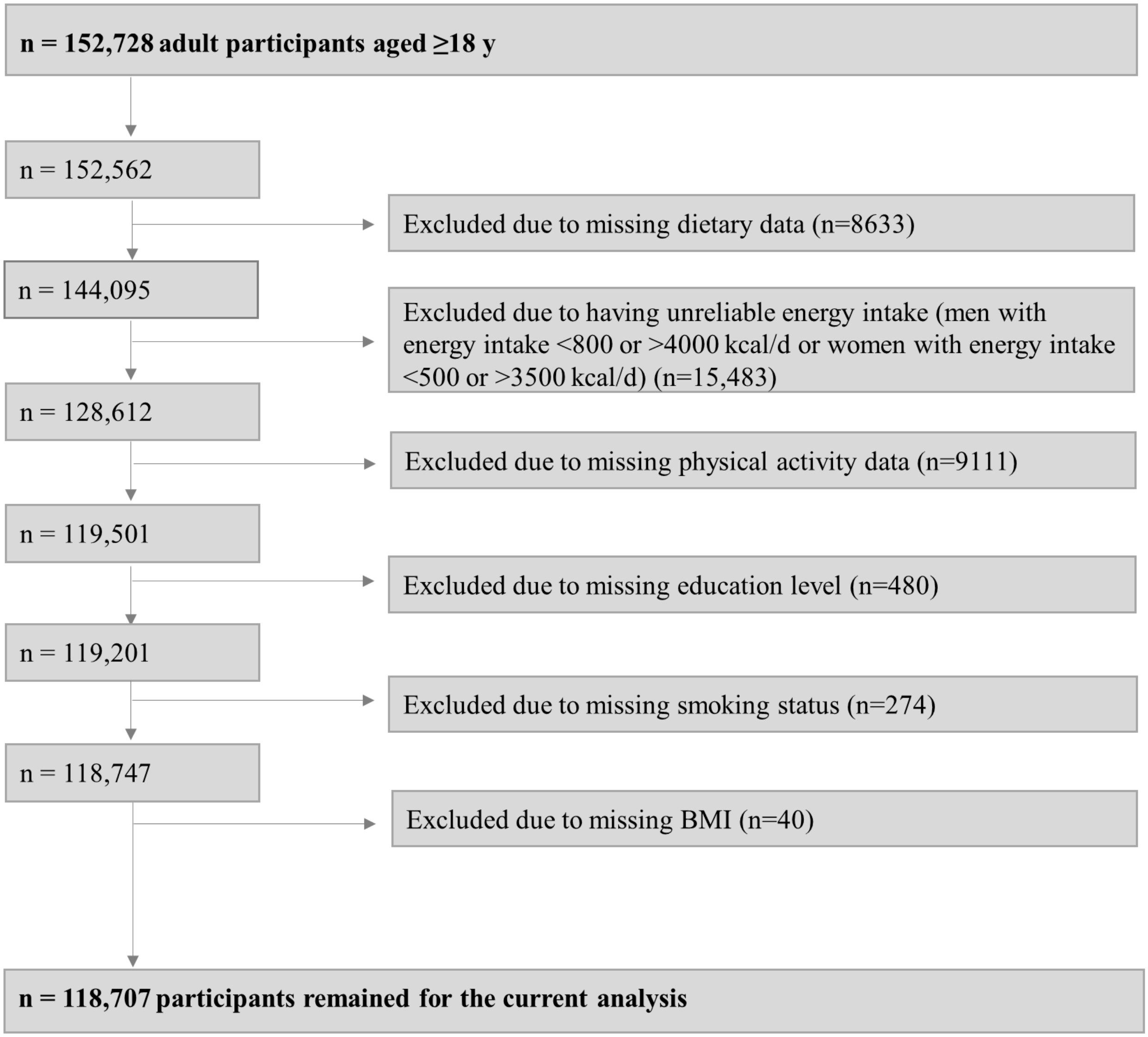

Supplemental Fig. 1 Flow chart of the exclusion criteria in Lifelines

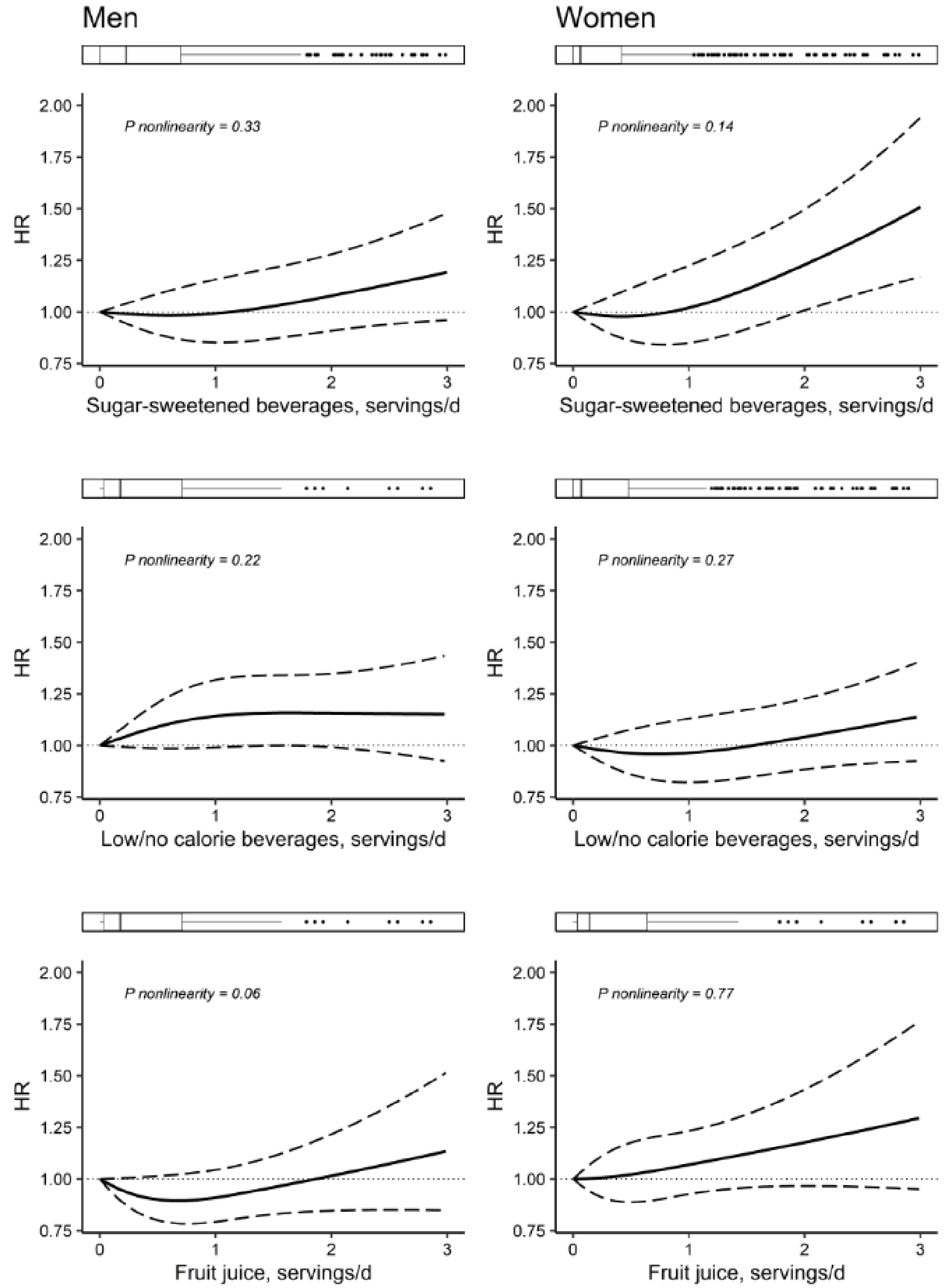

Supplemental Fig. 2 Dose-response associations between sugar-sweetened beverages, low/no calorie beverages and fruit juice consumption and all-cause mortality in men and women in Lifelines. Solid lines are risk estimates evaluated using restricted cubic splines indicating the shape of the association in the continuous scale Three knots with 0 serving/d as a reference value were placed. Beverages consumptions was truncated at 3 serving/d. Grey areas indicate 95% confidence intervals. Model was adjusted for age, education level, alcohol consumption, smoking status, moderate physical activity, sedentary behavior, baseline BMI, vegetables, fruit, meat and processed meat, coffee, tea, legumes, nuts, sugary foods, mutual adjustment for other beverages (sugar-sweetened beverages, low/no calorie beverages and fruit juice), grain, potatoes, fats, and energy intake.

Supplemental Table 1 Dietary consumption of 118,707 participants of Lifelines

Characteristics	All (n = 118,707)	Sex categories[1]	
		**Men (n= 47,943)**	**Women (n = 70,764)**
Grains, g/d	189 ± 80	224 ± 89	165 ± 64
Potatoes, g/d	86 [54-111]	97 [63-145]	71 [48-104]
Vegetables, g/d	108 [64-145]	85 [62-114]	108 [74-149]
Fruits, g/d	110 [42-220]	85 [42-220]	110 [42-220]
Meat and processed meat, g/d	77 ± 37	88 ± 39	70 ± 33
Coffee, ml/d	464 [232-581]	464 [348-697]	348 [161-464]
Tea, ml/d	232 [45-348]	89 [11-232]	232 [89-464]
Nuts, g/d	8 [3-16]	10 [4-21]	7 [3-14]
Legumes, g/d	11 [0-29]	16 [0-37]	11 [0-24]
Fats and oils, g/d	22 [12-32]	27 [15-38]	19 [10-27]
Sugary food, g/d	61 [37-93]	64 [39-98]	59 [36-89]

Value are in means ± SDs for normally distributes variables or medians [25th, 75th] for nonnormally distributed variables.

Supplemental Table 2 Baseline characteristics of 118,707 participants of Lifelines by categories of SSB consumption and stratified by sex

Characteristics	Men				Women			
	SSB consumption				SSB consumption			
	No consumption	>0-2 servings/wk	>2-<7 servings/wk	≥1 servings/d	No consumption	>0-2 servings/wk	>2-<7 servings/wk	≥1 servings/d
n	13,313	12,155	11,992	10,483	30,614	17,551	13,603	8996
Age, y	52 ± 12	47 ± 13	43 ± 12	39 ± 11	49 ± 12	44 ± 13	40 ± 12	36 ± 11
Education								
Low	595 (4)	386 (3)	359 (3)	420 (4)	1568 (5)	726 (4)	482 (3)	361 (4)
Moderate	7937 (60)	7076 (58)	7728 (64)	7626 (73)	20,146 (66)	10,874 (62)	9207 (68)	6489 (72)
High	4781 (36)	4693 (39)	3905 (33)	2437 (23)	8878 (29)	5951 (34)	3914 (29)	2146 (24)
Smoking status								
Never	4765 (36)	5410 (44)	5748 (48)	4788 (46)	13,324 (44)	9207 (52)	7363 (54)	4380 (49)
Former	6111 (46)	4382 (36)	3610 (30)	2388 (23)	12,213 (40)	5406 (31)	3395 (25)	1837 (20)
Current <10/d	1182 (9)	1170 (10)	1245 (10)	1204 (11)	2366 (8)	1569 (9)	1330 (10)	1022 (11)
Current ≥10/d	1255 (9)	1193 (10)	1389 (12)	2103 (20)	2711 (9)	1369 (8)	1515 (11)	1757 (20)
Moderate physical activity, MET-min/week	1926 [912-3492]	1776 [840-3150]	1692 [795-3008]	1470 [630-2765]	1680 [792-2940]	1542 [770-2715]	1466 [700-2571]	1278 [543-2426]
Sedentary behavior, min/week	840 [630-1260]	840 [630-1260]	840 [630-1260]	1050 [840-1260]	1050 [630-1260]	840 [630-1260]	841 [630-1260]	1050 [840-1470]
Alcohol use								
0 g/d	329 (3)	99 (1)	121 (1)	195 (2)	1437 (5)	347 (2)	394 (3)	356 (4)
>0-≤10 g/d	7487 (56)	7168 (59)	7472 (62)	6188 (59)	23,070 (75)	14,418 (82)	11,438 (84)	7519 (84)
>10-≤20 g/d	3604 (27)	3361 (28)	2996 (25)	2548 (24)	4971 (16)	2365 (14)	1501 (11)	923 (10)
>20 g/d	1893 (14)	1527 (12)	1403 (12)	1552 (15)	1136 (4)	421 (2)	270 (2)	198 (2)
Prevalent diabetes	929 (7)	218 (2)	144 (1)	81 (1)	1171 (4)	212 (1)	150 (1)	62 (1)
Hypercholesterolemia	3356 (25)	2080 (17)	1795 (15)	1104 (11)	4676 (15)	1660 (10)	984 (7)	491 (6)
Hypertension	3751 (28)	2412 (20)	2115 (18)	1542 (15)	8685 (28)	3666 (21)	2612 (19)	1425 (16)
History of CVD	682 (5)	372 (3)	316 (3)	220 (2)	665 (2)	269 (2)	174 (1)	127 (1)
BMI, kg/m^2	27.0 ± 3.8	26.0 ± 3.3	26.2 ± 3.6	26.1 ± 3.9	26.4 ± 4.7	25.1 ± 4.3	25.6 ± 4.8	25.4 ± 5.0
≥25	9362 (70)	7262 (60)	7393 (62)	6120 (58)	17,054 (56)	7663 (44)	6366 (47)	3946 (44)
SSB, serving/d	0	0.1 [0.1-0.2]	0.6 [0.4-0.7]	1.8 [1.3-2.7]	0	0.1 [0.1-0.2]	0.6 [0.4-0.7]	1.8 [1.3-2.5]
LNCB, serving/d	0.1 [0.0-1.3]	0.0 [0.0-0.1]	0.3 [0.0-0.7]	0.0 [0.0-0.6]	0.1 [0.0-0.7]	0.0 [0.0-0.1]	0.4 [0.0-0.9]	0.0 [0.0-0.7]
FJ, serving/d	0.1 [0.0-0.4]	0.2 [0.1-0.6]	0.3 [0.1-0.7]	0.3 [0.1-0.9]	0.1 [0.0-0.4]	0.2 [0.1-0.4]	0.3 [0.1-0.7]	0.4 [0.1-0.9]
Total energy, kcal/d	2114 ± 558	2273 ± 553	2396 ± 562	2587 ± 592	1691 ± 439	1836 ± 440	1933 ± 442	2079 ± 479
Grains, g/d	212 ± 90	229 ± 90	231 ± 87	225 ± 89	158 ± 65	172 ± 63	172 ± 60	166 ± 63
Potatoes, g/d	95 [55-137]	96 [63-143]	103 [66-148]	103 [71-148]	66 [41-103]	71 [50-104]	79 [55-108]	84 [56-111]
Vegetables, g/d	108 [63-149]	108 [62-147]	83 [62-113]	75 [42-113]	111 [75-160]	109 [75-150]	105 [64-113]	75 [61-112]

Fruits, g/d	110 [42-220]	110 [42-220]	85 [42-152]	76 [17-152]	152 [76-220]	110 [76-220]	85 [42-220]	76 [34-152]
Meat and processed meat, g/d	83 ± 40	84 ± 38	91 ± 38	96 ± 40	67 ± 34	69 ± 32	75 ± 31	76 ± 32
Coffee, ml/d	464 [348-697]	464 [348-697]	464 [348-697]	464 [232-697]	348 [232-581]	348 [161-464]	348 [89-464]	232 [0-464]
Tea, ml/d	116 [11-232]	116 [18-232]	89 [18-232]	45 [5-223]	232 [89-464]	232 [116-464]	232 [89-464]	161 [45-348]
Nuts, g/d	10 [3-21]	11 [5-21]	11 [5-21]	10 [3-21]	6 [2-14]	7 [3-14]	7 [3-14]	6 [2-13]
Legumes, g/d	16 [0-36]	19 [4-40]	17 [0-37]	14 [0-35]	11 [0-26]	11 [0-26]	9 [0-23]	7 [0-20]
Fats and oils, g/d	25 [14-36]	28 [17-38]	28 [17-39]	28 [15-39]	17 [8-26]	20 [11-28]	20 [11-28]	20 [11-28]
Sugary food, g/d	54 [31-84]	64 [40-94]	70 [45-104]	74 [45-113]	50 [29-77]	62 [40-90]	70 [45-101]	73 [46-108]

Value are in means ± SDs for normally distributes variables, medians [25th, 75th] for nonnormally distributed variables or *n* (%) for categorical variable. Abbreviation: CVD, cardiovascular disease; FJ, fruit juice; LNCB, low-calorie sweetened beverages; MET, metabolic equivalent task; SBB, sugar-sweetened beverages.

Supplemental Table 3 Baseline characteristics of 118,707 participants of Lifelines by categories of LNCB consumption and stratified by sex

**Characteristics**	**Men**				**Women**			
	**LNCB consumption**				**LNCB consumption**			
	**No consumption**	**>0-2 servings/wk**	**>2-<7 servings/wk**	**≥1 servings/d**	**No consumption**	**>0-2 servings/wk**	**>2-<7 servings/wk**	**≥1 servings/d**
n	22,027	8806	10,238	6872	30,348	15,036	15,256	10,124
Age, y	46 ± 13	48 ± 13	44 ± 13	43 ± 12	46 ± 13	46 ± 13	42 ± 13	41 ± 11
Education								
Low	928 (4)	278 (3)	320 (3)	234 (3)	1487 (5)	637 (4)	608 (4)	405 (4)
Moderate	14,123 (64)	5030 (57)	6598 (65)	4616 (67)	19,852 (65)	9557 (64)	10,260 (67)	7069 (70)
High	6976 (32)	3498 (40)	3320 (32)	2022 (30)	9009 (30)	4842 (32)	4388 (29)	2650 (26)
Smoking status								
Never	9246 (42)	3862 (44)	4673 (46)	2930 (43)	14,184 (47)	7565 (50)	7837 (51)	4688 (46)
Former	7439 (34)	3368 (38)	3393 (33)	2291 (33)	10,188 (34)	5112 (34)	4544 (30)	3007 (30)
Current <10/d	2206 (10)	842 (10)	1076 (10)	677 (10)	2539 (8)	1301 (9)	1425 (9)	1022 (10)
Current ≥10/d	3136 (14)	734 (8)	1096 (11)	974 (14)	3437 (11)	1058 (7)	1450 (10)	1407 (14)
Moderate physical activity, MET-min/week	1680 [740-3102]	1868 [914-3294]	1764 [840-3143]	1624 [735-2968]	1522 [710-2784]	1629 [828-2838]	1552 [753-2701]	1420 [640-2574]
Sedentary behavior, min/week	840 [630-1260]	840 [630-1260]	840 [630-1260]	1050 [840-1260]	840 [630-1260]	840 [630-1260]	945 [630-1260]	1050 [840-1470]
Alcohol use								
0 g/d	418 (2)	82 (1)	116 (1)	128 (2)	1311 (4)	334 (2)	452 (3)	437 (4)
>0-≤10 g/d	12,801 (58)	5248 (60)	6213 (61)	4053 (59)	23,626 (78)	12,154 (81)	12,579 (83)	8086 (80)
>10-≤20 g/d	5615 (26)	2421 (27)	2702 (26)	1771 (26)	4425 (15)	2145 (14)	1896 (12)	1294 (13)
>20 g/d	3193 (14)	1055 (12)	1207 (12)	920 (13)	986 (3)	403 (3)	329 (2)	307 (3)
Prevalent diabetes	408 (2)	245 (3)	307 (3)	412 (6)	573 (2)	308 (2)	341 (2)	373 (4)
Hypercholesterolemia	3511 (16)	1752 (20)	1707 (17)	1365 (20)	3572 (12)	1746 (12)	1475 (10)	1018 (10)
Hypertension	4185 (19)	1892 (22)	2134 (21)	1609 (23)	6886 (23)	3618 (24)	3448 (23)	2436 (24)
History of CVD	742 (3)	297 (3)	320 (3)	231 (3)	582 (2)	246 (2)	250 (2)	157 (2)
BMI, kg/m^2	25.9 ± 3.5	26.2 ± 3.4	26.6 ± 3.7	27.8 ± 4.1	25.2 ± 4.4	25.5 ± 4.3	26.2 ± 4.7	27.4 ± 5.4
≥25	12,749 (58)	5413 (62)	6775 (66)	5200 (76)	13,413 (44)	7240 (48)	8089 (53)	6287 (62)
SSB, serving/d	0.3 [0.0-1.3]	0.1 [0.0-0.2]	0.4 [0.0-0.9]	0.0 [0.0-0.9]	0.1 [0.0-0.7]	0.0 [0.0-0.1]	0.2 [0.0-0.6]	0.0 [0.0-0.6]
LNCB, serving/d	0	0.1 [0.1-0.2]	0.6 [0.4-0.7]	1.9 [1.3-2.5]	0	0.1 [0.1-0.2]	0.6 [0.4-0.7]	1.9 [1.3-2.5]
FJ, serving/d	0.2 [0.0-0.7]	0.2 [0.1-0.6]	0.3 [0.1-0.7]	0.2 [0.0-0.7]	0.1 [0.0-0.6]	0.1 [0.0-0.4]	0.2 [0.1-0.7]	0.2 [0.0-0.7]
Total energy, kcal/d	2355 ± 599	2236 ± 554	2339 ± 584	2346 ± 611	1822 ± 474	1787 ± 445	1847 ± 453	1842 ± 484
Grains, g/d	224 ± 91	226 ± 88	225 ± 87	219 ± 88	164 ± 66	168 ± 63	167 ± 61	160 ± 62
Potatoes, g/d	99 [63-147]	96 [61-142]	99 [64-146]	96 [63-142]	71 [46-104]	71 [48-103]	71 [52-104]	71 [46-104]
Vegetables, g/d	103 [62-114]	107 [62-127]	83 [62-113]	83 [62-113]	109 [74-151]	109 [76-150]	108 [74-114]	107 [63-114]

Fruits, g/d	85 [34-220]	110 [42-220]	85 [42-220]	85 [34-152]	110 [42-220]	152 [76-220]	110 [42-220]	85 [42-220]
Meat and processed meat, g/d	87 ± 40	83 ± 38	90 ± 37	95 ± 40	67 ± 34	69 ± 32	73 ± 31	76 ± 33
Coffee, ml/d	464 [348-697]	464 [348-697]	464 [348-697]	464 [348-697]	348 [161-464]	348 [232-581]	348 [161-464]	348 [45-581]
Tea, ml/d	89 [11-232]	116 [22-232]	89 [18-232]	54 [4-232]	232 [89-464]	232 [116-464]	232 [89-464]	232 [54-348]
Nuts, g/d	10 [3-21]	10 [5-20]	11 [5-21]	10 [4-21]	7 [2-14]	7 [3-14]	7 [3-14]	6 [2-14]
Legumes, g/d	16 [0-36]	20 [4-40]	18 [4-38]	16 [0-35]	11 [0-25]	11 [0-26]	9 [0-23]	7 [0-22]
Fats and oils, g/d	27 [16-39]	27 [16-38]	27 [16-38]	26 [13-37]	19 [10-27]	19 [10-27]	19 [10-27]	18 [9-27]
Sugary food, g/d	63 [38-99]	63 [39-94]	67 [42-100]	65 [39-100]	57 [34-87]	58 [36-86]	63 [41-93]	62 [37-95]

Value are in means ± SDs for normally distributes variables, medians [25th, 75th] for nonnormally distributed variables or *n* (%) for categorical variable.
Abbreviation: CVD, cardiovascular disease; FJ, fruit juice; LNCB, low-calorie sweetened beverages; MET, metabolic equivalent task; SBB, sugar-sweetened beverages.

Supplemental Table 4 Baseline characteristics of 118,707 participants of Lifelines by categories of FJ consumption and stratified by sex

**Characteristics**	**Men**				**Women**			
	**FJ consumption**				**FJ consumption**			
	**No consumption**	**>0-2 servings/wk**	**>2-<7 servings/wk**	**≥1 servings/d**	**No consumption**	**>0-2 servings/wk**	**>2-<7 servings/wk**	**≥1 servings/d**
n	10,881	14,263	16,425	6354	17,465	23,790	22,643	6866
Age, y	49 ± 13	46 ± 13	45 ± 13	41 ± 12	47 ± 13	44 ± 13	44 ± 13	40 ± 12
Education								
Low	581 (5)	465 (3)	496 (3)	218 (3)	1001 (6)	1004 (4)	871 (4)	261 (4)
Moderate	7702 (71)	8819 (62)	9738 (59)	4108 (65)	12,352 (71)	15,081 (64)	14,561 (64)	4744 (69)
High	2598 (24)	4979 (35)	6211 (38)	2028 (32)	4112 (23)	7705 (32)	7211 (32)	1861 (27)
Smoking status								
Never	3793 (35)	6224 (43)	7665 (47)	3029 (48)	7358 (42)	12,041 (51)	11,450 (51)	3425 (50)
Former	4422 (41)	5139 (36)	5274 (32)	1656 (26)	6548 (38)	7741 (32)	6851 (30)	1711 (25)
Current <10/d	936 (8)	1390 (10)	1726 (10)	749 (12)	1413 (8)	2018 (9)	2127 (9)	729 (10)
Current ≥10/d	1730 (16)	1510 (11)	1780 (11)	920 (14)	2146 (12)	1990 (8)	2215 (10)	1001 (15)
Moderate physical activity, MET-min/week	1752 [768-3265]	1756 [834-3096]	1728 [816-3110]	1630 [732-3018]	1477 [650-2766]	1542 [765-2730]	1575 [770-2760]	1505 [684-2764]
Sedentary behavior, min/week	1050 [840-1470]	840 [630-1260]	840 [630-1260]	840 [630-1260]	1050 [840-1470]	840 [630-1260]	840 [630-1260]	1050 [770-1260]
Alcohol use								
0 g/d	305 (3)	154 (1)	162 (1)	123 (2)	1115 (6)	604 (2)	576 (3)	239 (4)
>0-≤10 g/d	6056 (55)	8557 (60)	9875 (60)	3827 (60)	13,297 (76)	19,359 (81)	18,186 (80)	5603 (82)
>10-≤20 g/d	2791 (26)	3747 (26)	4374(27)	1597 (25)	2441 (14)	3213 (14)	3252 (14)	854 (12)
>20 g/d	1729 (16)	1805 (13)	2034 (12)	807 (13)	612 (4)	614 (3)	629 (3)	170 (3)
Prevalent diabetes	589 (5)	373 (3)	319 (2)	91 (1)	725 (4)	458 (2)	341 (2)	71 (1)
Hypercholesterolemia	2414 (22)	2528 (18)	2551 (16)	842 (13)	2495 (14)	2566 (11)	2252 (10)	498 (7)
Hypertension	2662 (25)	2968 (21)	3123 (19)	1067 (17)	4646 (27)	5452 (23)	4999 (22)	1291 (19)
History of CVD	534 (5)	482 (3)	436 (3)	138 (2)	411 (2)	358 (2)	360 (2)	106 (2)
BMI, kg/m^2	26.9 ± 3.9	26.2 ± 3.5	26.2 ± 3.6	26.2 ± 3.8	26.5 ± 5.0	25.6 ± 4.5	25.5 ± 4.5	25.5 ± 4.9
≥25	7400 (68)	8776 (62)	10,079 (61)	3882 (61)	9812 (56)	11,415 (48)	10,647 (47)	3155 (46)
SSB, serving/d	0.1 [0.0-0.7]	0.2 [0.0-0.7]	0.3 [0.0-0.9]	0.6 [0.1-1.4]	0.0 [0.0-0.1]	0.1 [0.0-0.3]	0.2 [0.0-0.6]	0.3 [0.0-1.3]
LNCB, serving/d	0.0 [0.0-0.6]	0.1 [0.0-0.5]	0.1 [0.0-0.6]	0.1 [0.0-0.7]	0.0 [0.0-0.6]	0.1 [0.0-0.5]	0.1 [0.0-0.6]	0.1 [0.0-0.7]
FJ, serving/d	0	0.1 [0.1-0.2]	0.6 [0.4-0.7]	1.7 [1.3-1.9]	0	0.1 [0.1-0.1]	0.6 [0.4-0.7]	1.9 [1.3-1.9]
Total energy, kcal/d	2217 ± 593	2276 ± 573	2363 ± 572	2545 ± 608	1674 ± 453	1795 ± 440	1898 ± 452	2051 ± 486
Grains, g/d	214 ± 90	224 ± 89	227 ± 88	230 ± 92	155 ± 66	167 ± 63	170 ± 61	171 ± 66
Potatoes, g/d	103 [64-148]	99 [63-146]	96 [63-140]	96 [61-144]	71 [43-103]	71 [48-103]	71 [51-104]	71 [50-104]
Vegetables, g/d	85 [62-114]	102 [62-114]	85 [62-114]	83 [62-114]	109 [74-150]	109 [75-150]	108 [74-114]	103 [63-114]

Fruits, g/d	85 [21-220]	85 [42-220]	85 [42-220]	85 [42-220]	152 [42-220]	127 [76-220]	110 [76-220]	85 [42-220]
Meat and processed meat, g/d	89 ± 40	87 ± 39	88 ± 38	91 ± 41	70 ± 34	70 ± 33	71 ± 32	72 ± 33
Coffee, ml/d	464 [348-697]	464 [348-697]	464 [348-697]	464 [232-697]	348 [232-581]	348 [161-464]	348 [161-464]	348 [54-464]
Tea, ml/d	80 [0-232]	116 [18-232]	89 [18-232]	89 [11-232]	232 [89-464]	232 [116-464]	232 [89-464]	232 [80-348]
Nuts, g/d	8 [3-19]	10 [5-21]	11 [5-21]	11 [5-21]	6 [2-13]	7 [3-14]	7 [3-14]	7 [3-15]
Legumes, g/d	15 [0-34]	18 [2-36]	18 [4-39]	18 [0-40]	7 [0-22]	11 [0-24]	11 [0-26]	11 [0-26]
Fats and oils, g/d	27 [15-38]	27 [17-38]	27 [15-38]	27 [14-39]	18 [8-26]	19 [10-27]	19 [11-27]	11 [0-28]
Sugary food, g/d	57 [33-91]	64 [40-96]	68 [42-101]	70 [43-107]	49 [28-79]	60 [37-88]	64 [41-94]	67 [42-102]

Value are in means ± SDs for normally distributes variables, medians [25th, 75th] for nonnormally distributed variables or *n* (%) for categorical variable.
Abbreviation: CVD, cardiovascular disease; FJ, fruit juice; LNCB, low-calorie sweetened beverages; MET, metabolic equivalent task; SBB, sugar-sweetened beverages.

Supplemental Table 5 Association between SSB, LNCB, and FJ consumption and all-cause mortality in 118,458 participants of Lifelines after omitting the first 2 years of follow-up

	HR (95%CI) by categories of beverages consumption				HR (95%CI) for each servings/d increment	*P* for interaction[1]
	No consumption	>0-2 servings/wk	>2-<7 servings/wk	≥1 servings/d		
**SSB**						
n	43,821	29,641	25,555	19,441	118,458	
Event (%)	1365 (3.1)	590 (2.0)	388 (1.5)	260 (1.3)	2603 (2.2)	
Model 3	1 (ref)	0.88 (0.80-0.98)	0.91 (0.80-1.02)	1.05 (0.90-1.22)	1.07 (1.00-1.13)	
Men						0.006
n	13,258	12,111	11,966	10,457	47,792	
Event (%)	658 (5.0)	312 (2.6)	227 (1.9)	168 (1.6)	1365 (2.9)	
Model 3	1 (ref)	0.77 (0.67-0.89)	0.82 (0.70-0.96)	1.01 (0.83-1.22)	1.04 (0.96-1.12)	
Women						
n	30,563	17,530	13,589	8984	70,666	
Event (%)	707 (2.3)	278 (1.6)	161 (1.2)	92 (1.0)	1238 (1.8)	
Model 3	1 (ref)	1.01 (0.88-1.17)	1.02 (0.85-1.22)	1.09 (0.86-1.39)	1.12 (1.02-1.23)	
**LNCB**						
n	52,241	23,790	25,461	16,966	118,458	
Event (%)	1337 (2.6)	513 (2.2)	454 (1.8)	299 (1.8)	2603 (2.2)	
Model 3[2]	1 (ref)	0.92 (0.83-1.03)	0.99 (0.89-1.10)	1.17 (1.03-1.33)	1.07 (1.02-1.13)	
Men						0.94
n	21,950	8774	10,215	6853	47,792	
Event (%)	693 (3.2)	256 (2.9)	251 (2.5)	165 (2.4)	1365 (2.9)	
Model 3	1 (ref)	0.91 (0.79-1.05)	1.01 (0.87-1.17)	1.20 (1.01-1.44)	1.07 (0.99-1.15)	
Women						
n	30,291	15,016	15,246	10,113	70,666	
Event (%)	644 (2.1)	257 (1.7)	203 (1.3)	134 (1.3)	1238 (1.8)	
Model 3	1 (ref)	0.93 (0.80-1.08)	0.95 (0.81-1.11)	1.090 (0.90-1.33)	1.06 (0.99-1.14)	
**FJ**						
n	28,261	37,983	39,015	13,199	118,458	
Event (%)	878 (3.1)	747 (2.0)	752 (1.9)	226 (1.7)	2603 (2.2)	
Model 3	1 (ref)	0.86 (0.78-0.95)	0.89 (0.80-0.98)	1.10 (0.95-1.29)	1.05 (0.97-1.12)	
Men						0.11
n	10,833	14,214	16,406	6339	47,792	
Event (%)	475 (4.4)	381 (2.7)	385 (2.3)	124 (2.0)	1365 (2.9)	
Model 3	1 (ref)	0.83 (0.72-0.95)	0.80 (0.70-0.923)	1.04 (0.85-1.28)	0.99 (0.89-1.10)	
Women						

n	17,428	23,769	22,609	6860	70,666
Event (%)	403 (2.3)	366 (1.5)	367 (1.6)	102 (1.5)	1238 (1.8)
Model 3	1 (ref)	0.90 (0.78-1.04)	0.98 (0.85-1.14)	1.14 (0.91-1.44)	1.09 (0.98-1.21)

Model 3: adjusted for total beverages, mutual adjustment for other beverages (LNCB or FJ), age, education level, alcohol consumption, smoking status, moderate physical activity, sedentary behavior, baseline BMI, grains, potatoes, vegetables, fruit, meat and processed meat, coffee, tea, legumes, nuts, fats and oils, sugary foods, mutual adjustment for other beverages (sugar-sweetened beverages, low/no calorie beverages and fruit juice) and energy intake.

Abbreviation: FJ, fruit juice; LNCB, low-calorie sweetened beverages; SBB, sugar-sweetened beverages.

[1] *P value* for interaction with sex was calculated in each serving/d increment for SSB and LNCB and using categorical model for FJ.

Supplemental Table 6 Association between SSB, LNCB, and FJ consumption and all-cause mortality in 118,707 participants of Lifelines when stratified by categories of BMI, education level, and prevalence of diseases

	**Categories of beverages consumption**				**Each servings/d**	***p* interaction**[1]
	**No consumption**	**>0-2 servings/wk**	**>2-<7 servings/wk**	**≥1 servings/d**		
**SSB**						
BMI						<.001
BMI <25, *n*/Event (%)	17,511/442 (2.5)	14,781/250 (1.7)	11,836/131 (1.1)	9413/100 (1.1)	53,541/923 (1.7)	
Model 3	1 (ref)	1.01 (0.86-1.18)	0.92 (0.75-1.14)	1.13 (0.88-1.45)	1.12 (1.02-1.23)	
BMI ≥25, *n*/Event (%)	26,416/1029 (3.9)	14,925/405 (2.7)	13,759/297 (2.2)	10,066/198 (2.0)	65,166/1929 (3.0)	
Model 3	1 (ref)	0.87 (0.77-0.98)	0.94 (0.82-1.08)	1.11 (0.93-1.32)	1.07 (1.00-1.15)	
Age						0.49
≤50, *n*/Event (%)	24,747/264 (1.1)	20,950/165 (0.8)	20,718/149 (0.7)	17,446/147 (0.8)	83,861/725 (1.0)	
Model 3	1 (ref)	0.82 (0.75-1.13)	0.88 (0.71-1.09)	1.06 (0.83-1.34)	1.10 (1.01-1.19)	
>50, *n*/Event (%)	19,180/1207 (6.3)	8756/490 (5.6)	4877/279 (5.7)	2033/151 (7.4)	34,846/2127 (6.1)	
Model 3	1 (ref)	0.87 (0.77-0.98)	0.94 (0.82-1.08)	1.11 (0.93-1.32)	1.08 (1.00-1.18)	
Education						0.27
Low/medium, *n*/Event (%)	30,268/1168 (3.8)	19,062/491 (2.6)	17,776/335 (1.9)	14,896/252 (1.7)	82,002/2241 (2.7)	
Model 3	1 (ref)	0.89 (0.80-1.00)	0.89 (0.79-1.02)	1.11 (0.95-1.11)	1.09 (1.02-1.16)	
High, *n*/Event (%)	13,659/308 (2.3)	10,644/164 (1.5)	7819/93 (1.2)	4583/46 (1.0)	36,705/611 (1.7)	
Model 3	1 (ref)	0.98 (0.80-1.20)	1.10 (0.86-1.41)	1.20 (0.85-1.69)	1.12 (0.98-1.28)	
Presence of diseases[2]						0.74
No, *n*/Event (%)	26,694/579 (2.2)	21,140/327 (1.5)	19,008/202 (1.1)	15,344/170 (1.1)	82,186/1278 (1.6)	
Model 3	1 (ref)	0.97 (0.84-1.12)	0.88 (0.74-1.05)	1.13 (0.93-1.38)	1.09 (1.01-1.17)	
Yes, *n*/Event (%)	17,233/892 (5.2)	8566/328 (3.8)	65857/226 (3.4)	4135/128 (3.1)	36,521/1574 (4.3)	
Model 3	1 (ref)	0.86 (0.76-0.99)	0.99 (0.85-1.16)	1.13 (0.92-1.38)	1.10 (1.01-1.20)	
**LNCB**						
BMI						0.80
BMI <25, *n*/Event (%)	26,213/529 (2.0)	11,189/200 (1.8)	10.630/130 (1.2)	5509/64 (1.2)	53,541/923 (1.7)	
Model 3	1 (ref)	1.04 (0.88-1.23)	1.02 (0.84-1.24)	1.15 (0.88-1.50)	1.10 (0.99-1.22)	
BMI ≥25, *n*/Event (%)	26,162/942 (3.6)	12,653/365 (2.9)	14,864/357 (2.4)	11,487/265 (2.3)	65,166/1929 (3.0)	
Model 3	1 (ref)	0.89 (0.78-1.00)	0.94 (0.83-1.07)	1.14 (0.99-1.31)	1.04 (0.98-1.10)	
Age						0.64
≤50, *n*/Event (%)	35,095/328 (0.9)	15,621/122 (0.8)	19,375/147 (0.8)	13,770/128 (0.9)	83,861/725 (1.0)	
Model 3	1 (ref)	0.94 (0.75-1.17)	0.94 (0.77-1.14)	1.03 (0.84-1.28)	1.01 (0.94-1.09)	
>50, *n*/Event (%)	17,280/1143 (6.6)	8221/443 (5.4)	6119/340 (5.6)	3226/201 (6.2)	34,846/2127 (6.1)	
Model 3	1 (ref)	0.93 (0.83-1.04)	0.97 (0.86-1.09)	1.22 (1.05-1.42)	1.04 (0.98-1.10)	
Education						0.90
Low/medium, *n*/Event (%)	36,390/1162 (3.2)	15,502/425 (2.7)	17,786/387 (2.2)	12,324/267 (2.2)	82,002/2241 (2.7)	

Model 3	1 (ref)	0.92 (0.82-1.03)	0.95 (0.84-1.07)	1.16 (1.01-1.33)	1.06 (1.00-1.12)	
High, *n*/Event (%)	15,985/309 (1.9)	8340/140 (1.7)	7708/100 (1.3)	4672/63 (1.3)	36,705/611 (1.7)	
Model 3	1 (ref)	0.97 (0.79-1.19)	0.99 (0.79-1.25)	1.11 (0.84-1.48)	1.05 (0.93-1.18)	
Presence of diseases[2]						0.18
No, *n*/Event (%)	36,700/701 (1.9)	16,186/251 (1.6)	17,865/196 (1.1)	11,435/130 (1.1)	82,186/1278 (1.6)	
Model 3	1 (ref)	0.93 (0.80-1.07)	0.83 (0.71-0.98)	1.01 (0.84-1.23)	1.01 (0.93-1.09)	
Yes, *n*/Event (%)	15,675/770 (4.9)	7656/314 (4.1)	7629/291 (3.8)	5561/199 (3.6)	36,521/1574 (4.3)	
Model 3	1 (ref)	0.94 (0.82-1.08)	1.07 (0.93-1.22)	1.24 (1.06-1.46)	1.08 (1.01-1.15)	
**FJ**						
BMI						0.61
BMI <25, *n*/Event (%)	11,134/288 (2.6)	17,862/261 (1.5)	18,362/295 (1.6)	6183/79 (1.3)	53,541/923 (1.7)	
Model 3	1 (ref)	0.82 (0.69-0.97)	0.97 (0.82-1.14)	1.10 (0.85-1.43)	1.08 (0.96-1.22)	
BMI ≥25, *n*/Event (%)	17,212/675 (3.9)	20,191/556 (2.8)	20,726/530 (2.6)	7037/168 (2.4)	65,166/1929 (3.0)	
Model 3	1 (ref)	0.87 (0.80-1.00)	0.87 (0.77-0.97)	1.12 (0.93-1.33)	1.04 (0.95-1.13)	
Age						0.36
≤50, *n*/Event (%)	17,861/191 (1.1)	26,518/202 (0.8)	28,486/218 (0.8)	10,996/114 (1.0)	83,861/725 (1.0)	
Model 3	1 (ref)	0.89 (0.73-1.08)	0.88 (0.72-1.08)	1.26 (0.99-1.60)	1.12 (1.01-1.24)	
>50, *n*/Event (%)	10,485/772 (7.4)	11,535/615 (5.3)	10,602/607 (5.7)	2224/133 (6.0)	34,846/2127 (6.1)	
Model 3	1 (ref)	0.86 (0.78-0.96)	0.89 (0.80-1.00)	0.99 (0.81-1.20)	0.99 (0.90-1.09)	
Education						0.48
Low/medium, *n*/Event (%)	21,636/818 (3.8)	25,369/628 (2.5)	25,666/612 (2.4)	9331/183 (2.0)	82,002/2241 (2.7)	
Model 3	1 (ref)	0.85 (0.76-0.94)	0.87 (0.79-0.97)	1.05 (0.89-1.25)	1.04 (0.96-1.12)	
High, *n*/Event (%)	6710/145 (2.2)	12,684/189 (1.5)	13,422/213 (1.6)	3889/64 (1.6)	36,705/611 (1.7)	
Model 3	1 (ref)	0.96 (0.77-1.20)	0.98 (0.79-1.22)	1.28 (0.94-1.75)	1.08 (0.94-1.25)	
Presence of diseases[2]						0.95
No, *n*/Event (%)	17,959/386 (2.1)	26,430/370 (1.4)	27,903/398 (1.4)	9894/124 (1.3)	82,186/1278 (1.6)	
Model 3	1 (ref)	0.88 (0.76-1.02)	0.93 (0.81-1.07)	1.12 (0.91-1.39)	1.07 (0.97-1.18)	
Yes, *n*/Event (%)	10,387/577 (5.6)	11,623/447 (3.8)	11,185/427 (3.8)	3326/123 (3.7)	36,521/1574 (4.3)	
Model 3	1 (ref)	0.86 (0.76-0.98)	0.88 (0.78-1.00)	1.08 (0.88-1.33)	1.03 (0.94-1.14)	

Model 3: adjusted for total beverages, mutual adjustment for other beverages (LNCB or FJ), age, education level, alcohol consumption, smoking status, moderate physical activity, sedentary behavior, baseline BMI, grains, potatoes, vegetables, fruit, meat and processed meat, coffee, tea, legumes, nuts, fats and oils, sugary foods, mutual adjustment for other beverages (sugar-sweetened beverages, low/no calorie beverages and fruit juice) and energy intake.
Abbreviation: FJ, fruit juice; LNCB, low-calorie sweetened beverages; SBB, sugar-sweetened beverages.
[1] *P value* for interaction with sex was calculated in each serving/d increment for SSB and LNCB and using categorical model for FJ.
[2] Presence of diseases: prevalence of self-reported diabetes or history of hypertension, hypercholesterolemia or CVD at baseline.

Supplemental Table 7 Association between SSB, LNCB, and FJ consumption and all-cause mortality among daily consumers of SSB, LNCB and FJ in Lifelines.

	HR (95%CI) for each servings/d among daily consumers
SSB	1.13 (1.02-1.25)
LNCB	0.98 (0.84-1.14)
FJ	0.98 (0.83-1.15)

Model 3: adjusted for total beverages, mutual adjustment for other beverages (LNCB or FJ), age, education level, alcohol consumption, smoking status, moderate physical activity, sedentary behavior, baseline BMI, grains, potatoes, vegetables, fruit, meat and processed meat, coffee, tea, legumes, nuts, fats and oils, sugary foods, and energy intake.
Abbreviation: FJ, fruit juice; LNCB, low-calorie sweetened beverages; SBB, sugar-sweetened beverages.
